# Supplementary material for: Direct probing of phonon mode specific electron–phonon scatterings in two-dimensional semiconductor transition metal dichalcogenides
Source: Nat Commun. 2021 Jul 26;12:4520. doi: 10.1038/s41467-021-24875-2 (PMC8313722; doi:10.1038/s41467-021-24875-2)
Supplement: Supplementary file 1 — Supplementary Information [file 41467_2021_24875_MOESM1_ESM.pdf]

Supplementary Information

Direct probing of phonon mode specific electron–phonon scatterings in two-dimensional semiconductor transition metal dichalcogenides

Duk Hyun Lee[1§], Sang-Jun Choi[2§], Hakseong Kim[1], Yong-Sung Kim[1] and Suyong Jung[1*]

[1]Korea Research Institute of Standards and Science, Daejeon 34113, Korea (Republic of)

[2]Institute for Theoretical Physics and Astrophysics, University of Wűrzburg, D-97074 Wűrzburg, Germany

[§] These authors contributed equally to this work.

* e-mail address : syjung@kriss.re.kr

Supplementary Figures

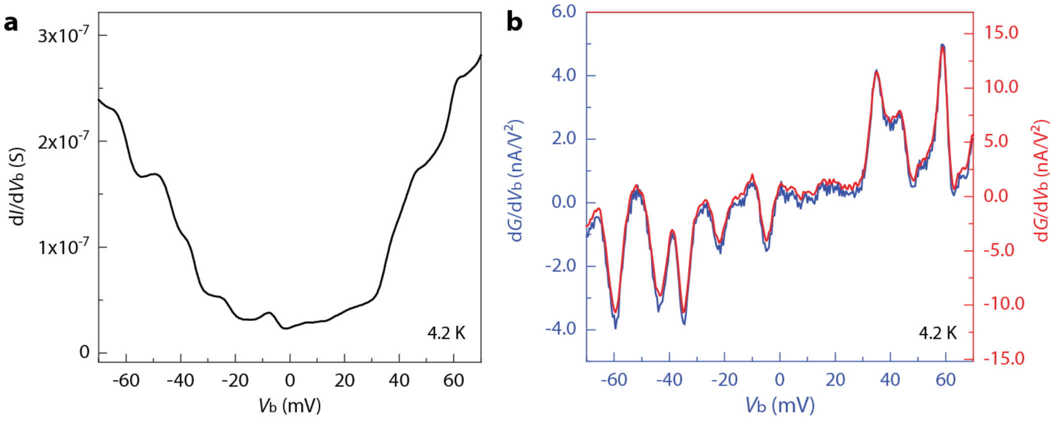

Supplementary Figure 1. Inelastic electron tunnel spectroscopy measurement in graphite–SC-TMD–graphite vertical tunnel junctions. a, b, Inelastic tunnel electron spectra represented in dI/dV_b – V_b (**a**) and dG/dV_b – V_b (**b**) from one of the bilayer WSe_2 tunnel devices. The solid red line in Supplementary Figure 1**b** is obtained by numerically differentiating the dI/dV_b – V_b spectrum (**a**) measured with a primary AC lock-in amplifier with frequency f (43.33 Hz), and the solid blue line is independently measured with a secondary AC lock-in synchronized at a frequency of 2f. All spectra were measured at T = 4.2 K with an excitation voltage of 1 mV.

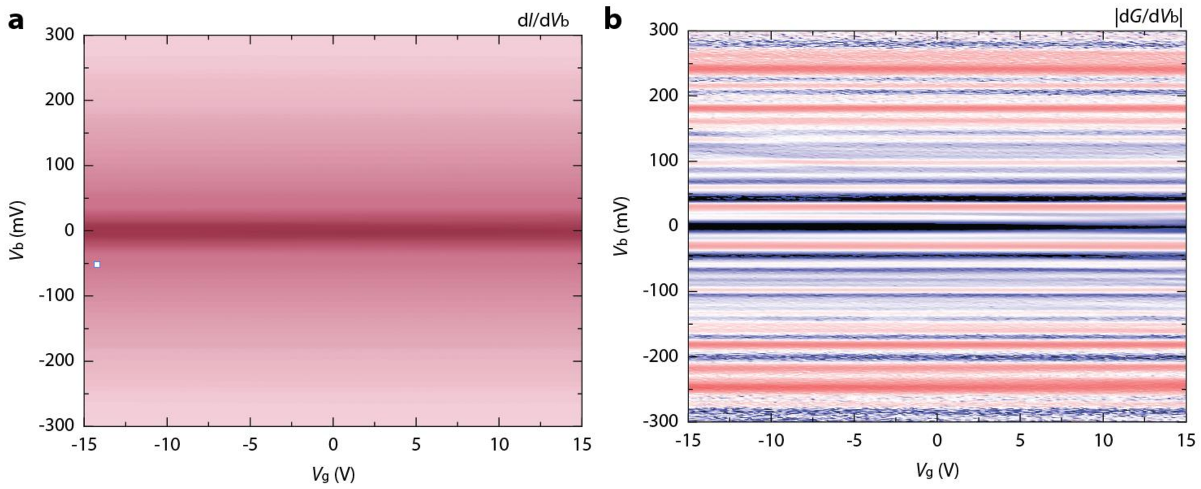

Supplementary Figure 2. Two-dimensional gate mappings of inelastic electron tunnel spectra. **a, b,** High resolution two-dimensional dI/dV_b (**a**) and |dG/dV_b| (**b**) gate mappings composed of 121 independent spectra measured at varying V_g in steps of ΔV_g = 0.25 V for one of the monolayer WSe_2 vertical junctions. No visible V_g-dependent tunnel spectra are observed. The high-energy ($|V_b|$ > 100 mV) IETS dG/dV_b features can be related to the phonons of the graphite electrodes and the two-phonon excitations of the monolayer WSe_2 and the graphite.

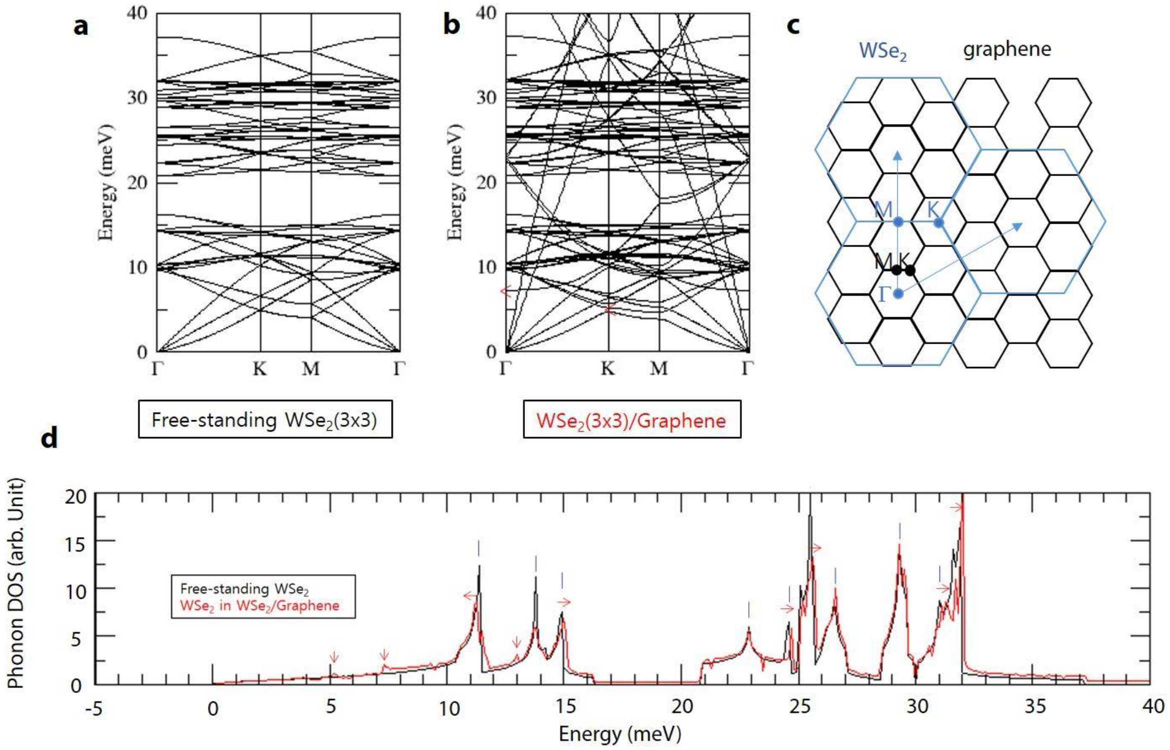

Supplementary Figure 3. First-principle calculations for phonons in WSe_2 and WSe_2–graphene heterostructures. **a, b** Zone-folded phonon dispersion relations of free-standing WSe_2(3×3) (**a**) and a WSe_2(3×3)–graphene(4×4) heterostructure (**b**). **c**, Schematic representation of the lattice alignment in the WSe_2(3×3)–graphene(4×4) heterostructure with a zero twist angle. **d**, Phonon density of states (DOS) of the free-standing WSe_2 (black line) and WSe_2(3×3)–graphene(4×4) heterostructure (red line). The phonon DOS shifts in the WSe_2(3×3)–graphene(4×4) sample are marked with red arrows.

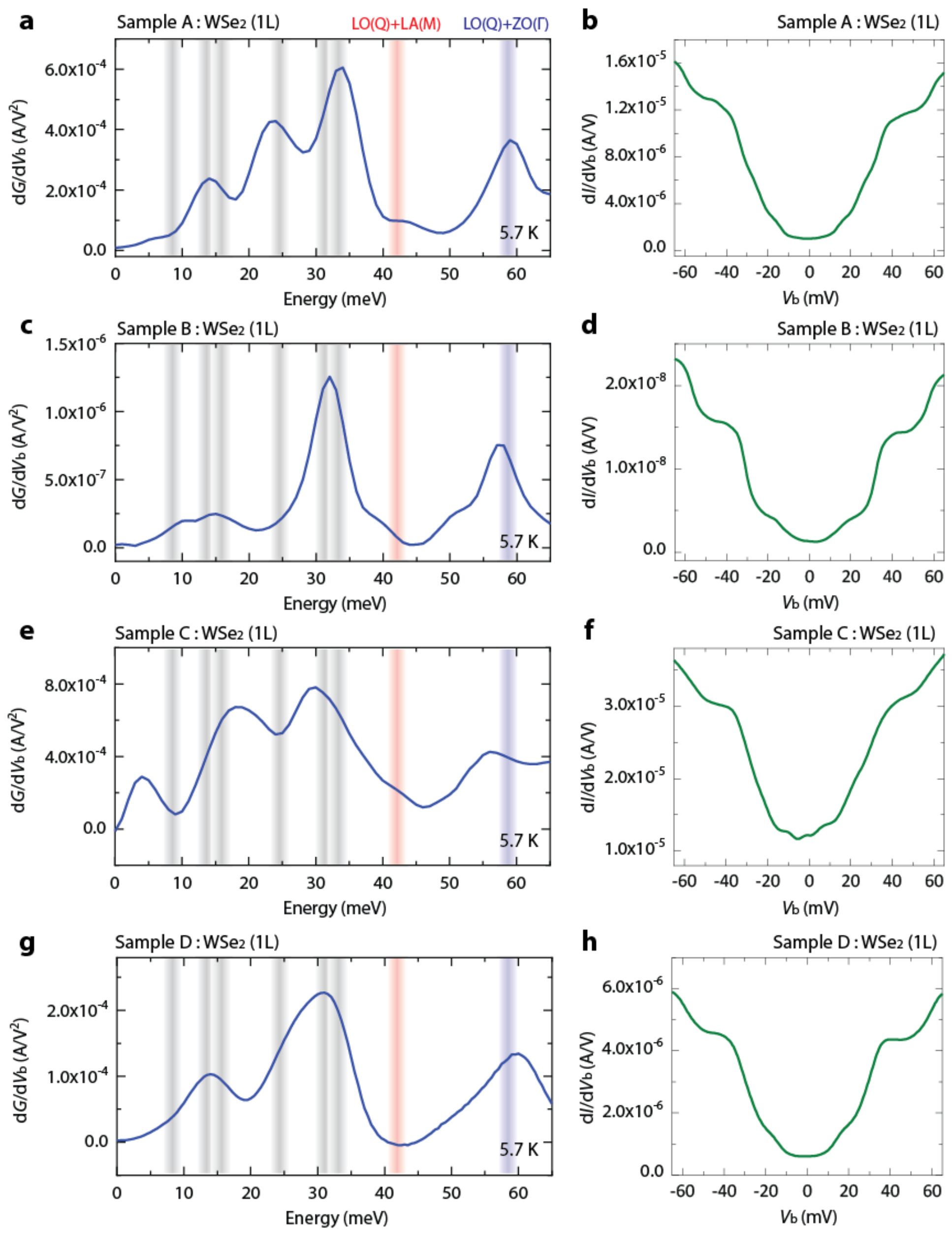

Supplementary Figure 4. Inelastic electron tunnel spectroscopy measurements in monolayer WSe_2 tunnel junctions. (**a–h**) Series of dG/dV_b and dI/dV_b spectra from a set of four monolayer WSe_2 tunnel devices, sample A to sample D. All spectra were measured at T = 5.7 K with an excitation voltage of 1 mV. Sample D is discussed in the main text.

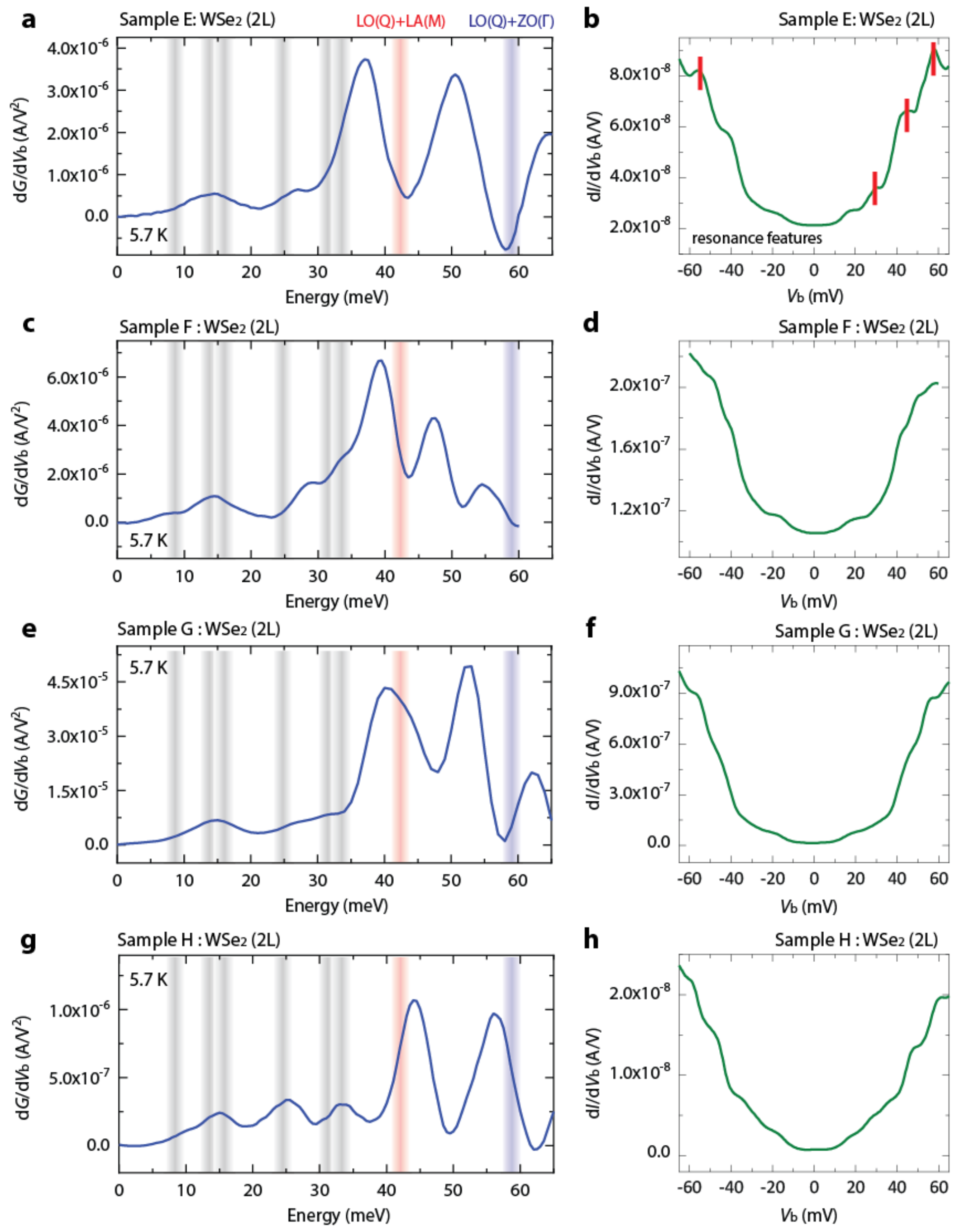

Supplementary Figure 5. Inelastic electron tunnel spectroscopy measurements in bilayer WSe_2 tunnel junctions. (**a–h**) Series of dG/dV_b and dI/dV_b spectra from a set of four bilayer WSe_2 tunnel devices, sample E to sample H. All spectra were measured at T = 5.7 K with an excitation voltage of 1 mV. Sample H is discussed in the main text. Sample E reveal several dI/dV_b resonance features that distort the dG/dV_b spectra for phonon assignments.

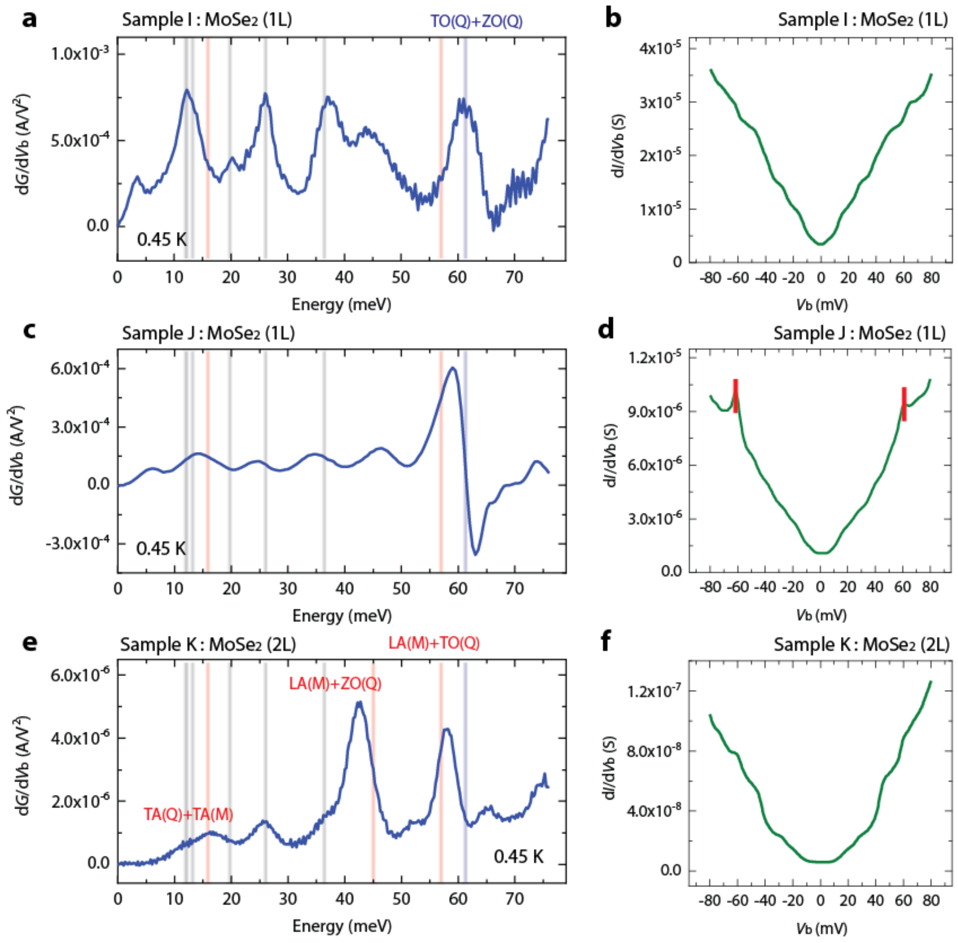

Supplementary Figure 6. Inelastic electron tunnel spectroscopy measurements in mono-and bilayer $MoSe_2$ tunnel junctions. (**a**–**f**) Series of dG/dV_b and dI/dV_b spectra from an additional set of mono- and bilayer $MoSe_2$ tunnel devices that are not discussed in the main text. All spectra were measured at T = 0.45 K with an excitation voltage of 0.5 mV.

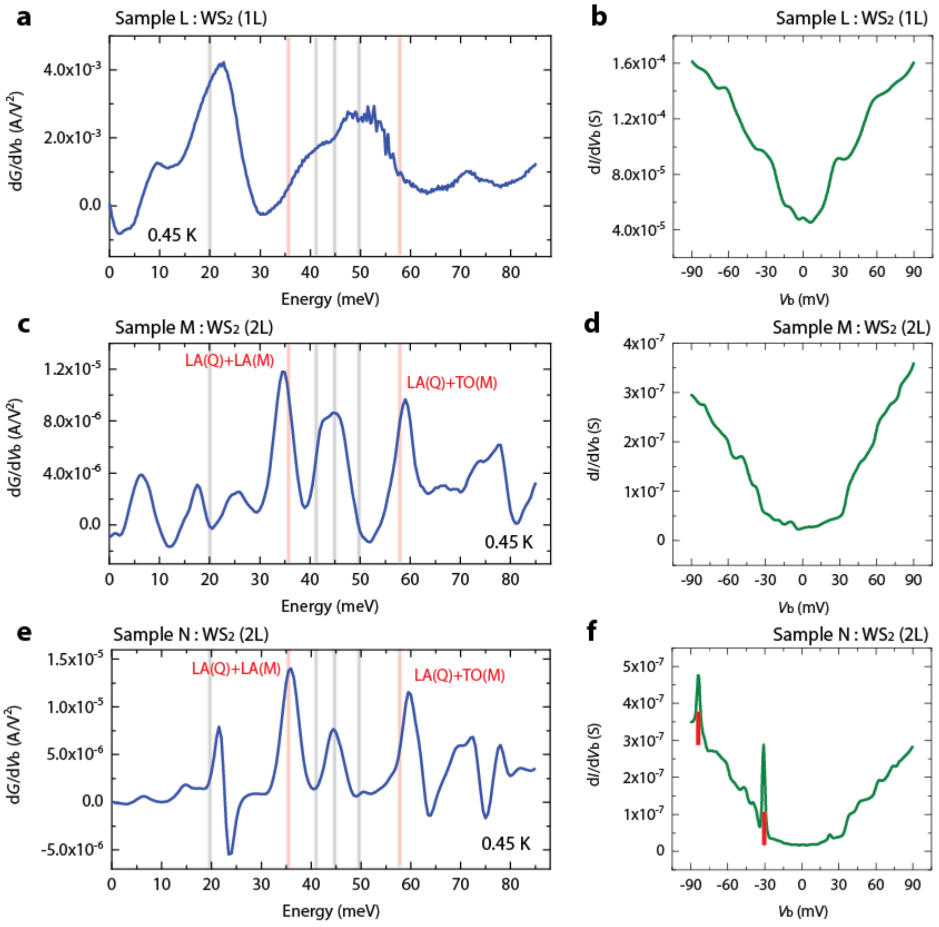

Supplementary Figure 7. Inelastic electron tunnel spectroscopy measurements in mono-and bilayer WS_2 tunnel junctions. (**a–f**) Series of dG/dV_b and dI/dV_b spectra from an additional set of mono- and bilayer WS_2 tunnel devices that are not discussed in the main text. All spectra were measured at T = 0.45 K with an excitation voltage of 0.5 mV.

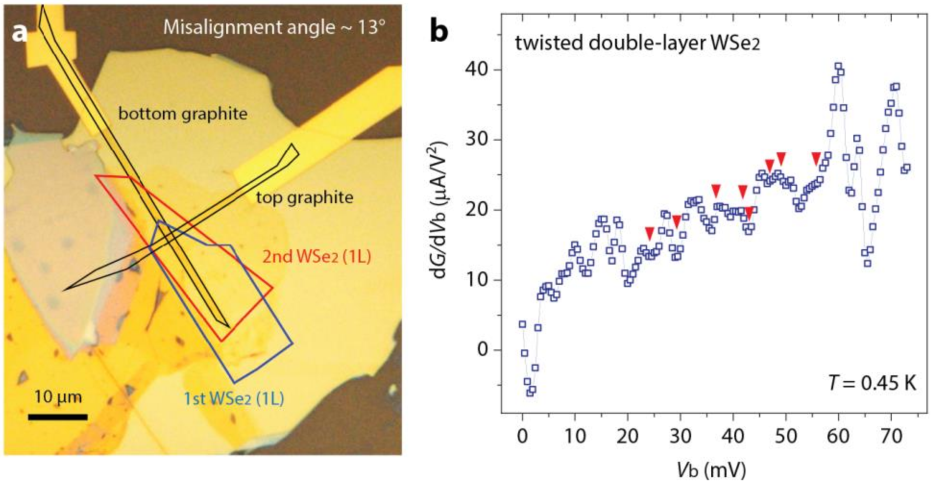

Supplementary Figure 8. Inversion-symmetry broken, double-layer WSe_2 junction. a, Optical viewgraph of a twisted double layer WSe_2 planar tunnel junction. The misalignment angle between the first and second WSe_2 monolayers, as judged from the crystallographic angles of each flake, is estimated to be around 13°. **b,** IETS $dG/dV_b - V_b$ spectrum from the twisted double layer WSe_2 device, measured at T = 0.45 K with an excitation voltage of 0.5 mV. The inverted red triangles indicate the energies of all plausible two-phonon combinations with Q and M phonons, at which strong IETS features can be expected in the conventional Bernal stacked WSe_2 bilayers.

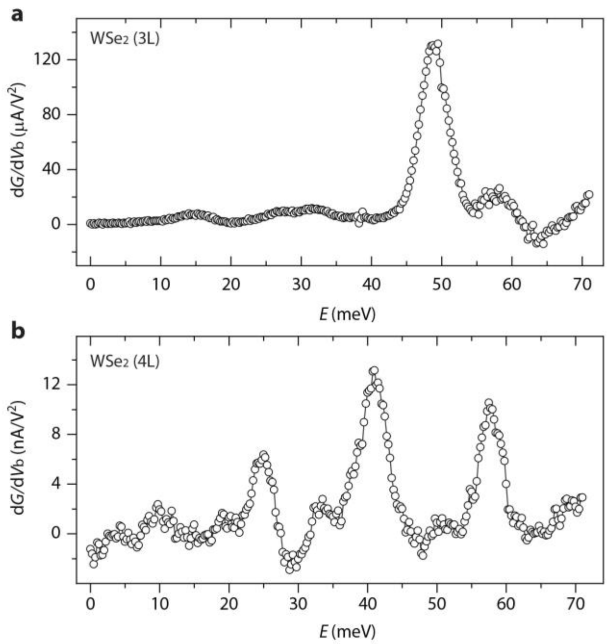

Supplementary Figure 9. Inelastic electron tunnel spectroscopy measurements in multi-layer WSe_2 tunnel junctions. a, b, Series of dG/dV_b and dI/dV_b spectra from three-layer (**a**) and four-layer (**b**) WSe_2 tunnel devices. All spectra were measured at T = 0.45 K with an excitation voltage of 0.3 mV.

Supplementary Note

Theoretical descriptions on the vertical tunneling of graphite–SC-TMD–graphite junctions

We theoretically describe the vertical quantum tunnel events with the following Hamiltonian, $H = H_{\mathrm{e}} + H_{\mathrm{ph}} + H_{\mathrm{int}}$. Electrons in SC-TMD films, and source and drain graphite electrodes are described by the Hamiltonian $H_{\mathrm{e}} = \sum_{\kappa} \epsilon_{\kappa} c_{\kappa}^{\dagger} c_{\kappa} + \sum_{p,\nu=L,R} \epsilon_{pL} d_{p\nu}^{\dagger} d_{p\nu} + \sum_{\substack{\nu=L,R,\\ p,\kappa}} V_{p\kappa\nu} \left(d_{p\nu}^{\dagger} c_{\kappa} + c_{\kappa}^{\dagger} d_{p\nu} \right)$. The operator $c_{\kappa}^{\dagger}$ (c_{κ}) creates (annihilates) an electron in the SC-TMD at the κ ($K, K', Q_1, Q_2, Q_3, Q_4, Q_5, Q_6$) valley with conduction band edge energy ϵ_{κ}. Since the electrons at the bottom of the conduction band edges mostly participate in quantum tunneling events, we do not take into account the full dispersion of each valley for simplicity. The operator $d_{p\nu}^{\dagger}$ ($d_{p\nu}$) creates (annihilates) an electron with momentum p in the source $\nu = L$ and drain $\nu = R$ electrodes, coupled by hopping matrix elements $V_{p\kappa\nu}$. The phonons in the SC-TMD film are depicted by the Hamiltonian $H_{\mathrm{ph}} = \sum_{\lambda,q} \hbar\omega_{\lambda q} a_{\lambda q}^{\dagger} a_{\lambda q}$, where the subscript λ denotes each phonon mode with frequency $\omega_{\lambda q}$ and momentum q at the high-symmetry points which connect the momenta of electrons at the conduction band edges of the K and Q valleys. We confine the electron–phonon interactions within the SC-TMD by $H_{\mathrm{int}} = \sum_{q} M_{\kappa+q,\kappa}^{\lambda} c_{\kappa+q}^{\dagger} c_{\kappa} a_{\lambda q} + h.c.$. Generally, the electron–phonon coupling matrix element is evaluated by $M_{\kappa+q,\kappa}^{\lambda} = \langle \kappa + q | \delta V_{q\lambda} | \kappa \rangle$, where $\delta V_{q\lambda}$ is the derivative of the effective potential generated from the displacement of atomic arrangements by the phonon mode λ, and $|\kappa\rangle$ is the electronic state with crystal momentum κ.

We highlight that special attention to $M^{\lambda}_{\kappa+q,\kappa}$ is required for the systems with spin-momentum locking, since the spin states allocated to the electronic states with momenta κ and $\kappa + q$ are different. In a system with the spin-momentum locking, therefore, we need to treat the electronic wave function $\langle \boldsymbol{r}|\kappa\rangle = \phi_{\kappa}(\boldsymbol{r})\eta_{\kappa}$ with the momentum-dependent spinor η_{κ} and spatial wave function $\phi_{\kappa}(\boldsymbol{r})$, such that

$$M^{\lambda}_{\kappa+q,\kappa} = \eta^{\dagger}_{\kappa+q}\eta_{\kappa}\int d\boldsymbol{r}\phi^{*}_{\kappa+q}(\boldsymbol{r})\delta V_{q\lambda}(\boldsymbol{r})\phi_{\kappa}(\boldsymbol{r}). \quad (1)$$

Note that the pre-factor $\eta^{\dagger}_{\kappa+q}\eta_{\kappa}$ appearing in $M^{\lambda}_{\kappa+q,\kappa}$ gives rise to the geometric phase, while the pre-factor becomes $\eta^{\dagger}_{\kappa+q}\eta_{\kappa} = 1$ in systems without spin-momentum locking. The quantal phase of the pre-factor has a close connection to the geometric interpretation, *i.e.*, $\arg\left(\eta^{\dagger}_{\kappa+q}\eta_{\kappa}\right) = \int_{C} d\boldsymbol{R}\cdot\eta^{\dagger}(\boldsymbol{R})\boldsymbol{\nabla}_{\boldsymbol{R}}\eta(\boldsymbol{R})$, as a line integral of the Berry connection along the geodesic C connecting the spin states of $\eta_{\kappa+q}$ and η_{κ} on the Bloch sphere. Once the combined geodesic lines form a closed geodesic polygon, the total quantal phase factor becomes gauge-independent and physically detectable.

Theoretical evaluation of the interacting two-particle Green function

Here, we theoretically evaluate the two-particle Green function $G_{\kappa\kappa'}(\tau, s, t)$ of the conducting electrons that interact with phonons up to the second order of electron–phonon coupling strength g $(g < 1)$. First, we briefly introduce many-body perturbation theory $G_{\kappa\kappa'}(\tau, s, t)$ with Feynman diagrams. We combine the parametric terms in the perturbative expansions of $G_{\kappa\kappa'}(\tau, s, t)$ according to the dG/dV_b peak order in the main text, *i.e.*, $G_{\kappa\kappa'}(\tau, s, t) \sim G^{0}_{\kappa\kappa'}(\tau, s, t) + G^{1}_{\kappa\kappa'}(\tau, s, t) + G^{2}_{\kappa\kappa'}(\tau, s, t)$. Next, we evaluate $G^{1}_{\kappa\kappa'}(\tau, s, t)$ that is responsible for the first-order dG/dV_b peaks. Last, we demonstrate that quantum interference with a consideration

of the geometric phase plays an important role in $G^2_{\kappa\kappa'}(\tau, s, t)$, which is attributed to the second-order peaks in inelastic electron tunnel features dG/dV_b.

Perturbative expansion of $G_{\kappa\kappa'}(\tau, s, t)$ and Feynman diagrams

The perturbative expansion of the two-particle Green function in the interaction picture is

$$G_{\kappa\kappa'}(\tau, s, t) = \Theta(s)\Theta(t)\langle \hat{c}_\kappa(\tau - s)U(\tau - s, \tau)\hat{c}^\dagger_{\kappa'}(\tau)\hat{c}_{\kappa'}(t)U(t, 0)\hat{c}^\dagger_\kappa(0)\rangle, \tag{2}$$

where Θ is the Heaviside theta function. The operators $\hat{c}_\kappa$ ($\hat{c}^\dagger_\kappa$) with the hat are defined in the interaction picture $\hat{c}_\kappa(t) = e^{\frac{iH_0 t}{\hbar}} c_\kappa(0) e^{-\frac{iH_0 t}{\hbar}}$. We divide the full Hamiltonian $H = H_0 + H_{int}$ into a bare Hamiltonian $H_0 = H_\mathrm{e} + H_\mathrm{ph}$ and an interaction Hamiltonian $\hat{H}_{int} = \sum_q M^\lambda_{\kappa+q,\kappa}\hat{c}^\dagger_{\kappa+q}\hat{c}_\kappa\hat{a}_{\lambda q} + h.c.$, where $\hat{a}_{\lambda q} = a_{\lambda q}e^{-i\omega_{\lambda q}t}$. Here, $U(t_b, t_a)$ is the time-evolution operator expressed in

$$U(t_b, t_a) = 1 + \left(-\frac{i}{\hbar}\right)\int_{t_a}^{t_b} dt'\hat{H}_{int}(t') + \left(-\frac{i}{\hbar}\right)^2 \int_{t_a}^{t_b} dt' \int_{t_a}^{t'} dt''\hat{H}_{int}(t')\hat{H}_{int}(t'') + \cdots, \tag{3}$$

where $\hat{H}_{int}$ is given in the interaction picture. For convenience, we denote the second and third terms in the right-hand side as $u_1(t_b, t_a)$ and $u_2(t_b, t_a)$, respectively. In Supplementary Figure 10, we provide Feynman diagrams representing each term in the perturbative expansion of $G_{\kappa\kappa'}(\tau, s, t) \sim G^0_{\kappa\kappa'}(\tau, s, t) + G^1_{\kappa\kappa'}(\tau, s, t) + G^2_{\kappa\kappa'}(\tau, s, t)$ up to the second order of electron–phonon strength $g \sim \left|M^\lambda_{\kappa',\kappa}\right|^2$. We note that $G_{\kappa\kappa'}(\tau, s, t)$ in the time domain becomes the transmission probability in the energy domain, and thus, $G_{\kappa\kappa'}(\tau, s, t)$ includes not only the propagating amplitudes of the electrons in the conduction band but also those of the holes, which correspond to the complex conjugates of the electron tunneling amplitude. We have combined the

Feynman diagrams in accordance with the number of phonons exchanged between electrons and holes in the conduction band. We assume no available phonons exist for conducting electrons to absorb at T < 65 K since $k_{\mathrm{B}}T \ll \min\{\hbar\omega_{\lambda q}\}$; we note that the subscript λ denotes the mode of the phonons with frequency $\omega_{\lambda q}$ and momentum q at high-symmetry points in the main text.

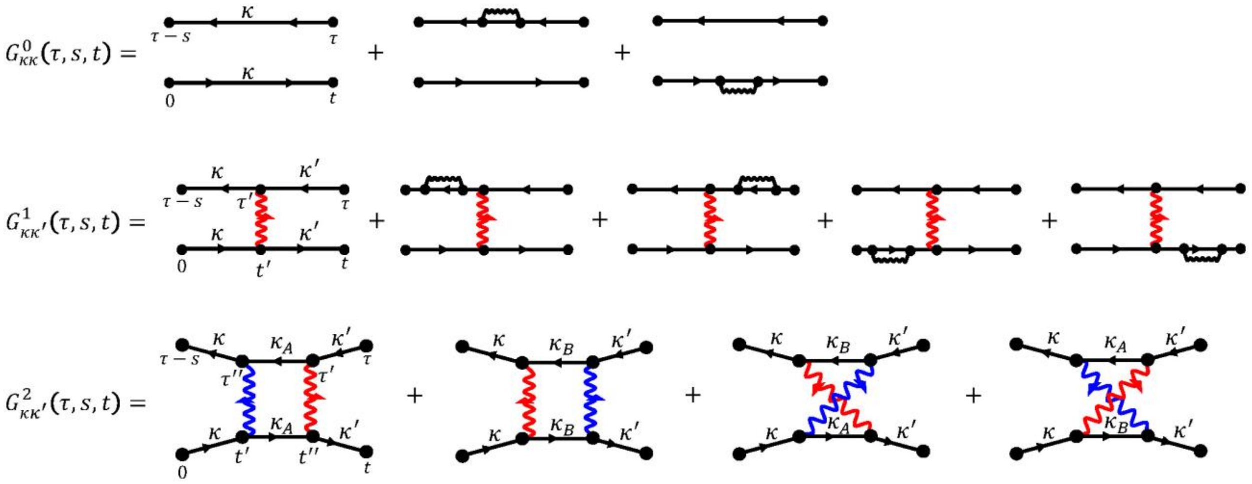

Supplementary Figure 10. Feynman diagrams for the perturbative expansion of $\boldsymbol{G_{\kappa\kappa'}(\tau, s, t) \sim G^0_{\kappa\kappa'}(\tau, s, t) + G^1_{\kappa\kappa'}(\tau, s, t) + G^2_{\kappa\kappa'}(\tau, s, t)}$. The solid lines with right arrows propagating forward in the time domain represent electron scattering processes. The solid lines with left arrows represent holes moving backward in the time domain, corresponding to the complex conjugation of the tunnel probability. The wavy lines indicate the propagations of phonons. Specifically, the colored wavy lines indicate the phonon exchanges between electrons and holes, while differing colors indicating different phonon modes such as LA and TO.

$G^0_{\kappa\kappa'}$ is responsible for the elastic electron tunneling. $G^1_{\kappa\kappa'}(\tau, s, t)$, which is attributed to the first-order dG/dV_{b} peaks, explains the electron tunneling processes that exchange a single phonon between electrons and holes in the conduction band. Electrons (holes) can be scattered by other phonons up to the fifth terms in $G^1_{\kappa\kappa'}(\tau, s, t)$ (Supplementary Figure 10). In the perturbative

expansion of $G^{1}_{\kappa\kappa'}(\tau, s, t)$, the propagations with dressing phonons are on the order of g^2, while those without dressing phonons are on the order of g. Actually though, we find out that dressing phonons change the electron tunneling little, when compared to an electron tunneling process without dressing phonons. Finally, $G^{2}_{\kappa\kappa'}(\tau, s, t)$ corresponds to the electron tunneling processes exchanging two phonons between electrons and holes in the conduction band, with $G^{2}_{\kappa\kappa'}(\tau, s, t)$ being responsible for the second-order peaks in dG/dV_b.

Evaluation of $G^{1}_{\kappa\kappa'}(\tau, s, t)$ for the first-order peaks in d*G*/d*V*b

We evaluate $G^{1}_{\kappa\kappa'}(\tau, s, t)$ according to the Feynman diagrams in Supplementary Figure 10 and provide the related transmission probability. We begin with an evaluation of the first term of $G^{1}_{\kappa\kappa'}(\tau, s, t) = \Theta(s)\Theta(t)\langle \hat{c}_{\kappa}(\tau - s) u_1(\tau - s, \tau) \hat{c}^{\dagger}_{\kappa'}(\tau) \hat{c}_{\kappa'}(t) u_1(t, 0) \hat{c}^{\dagger}_{\kappa}(0)\rangle$. We note that $G^{1}_{\kappa\kappa'}(\tau, s, t)$ can be understood as a product of electron and hole Green functions as $G^{1}_{\kappa\kappa'}(\tau, s, t) = G^{1,h}_{\kappa\kappa'}(\tau, s) G^{1,e}_{\kappa\kappa'}(t)$, where $G^{1,e}_{\kappa\kappa'}(t) = -i\Theta(t)\langle \hat{c}_{\kappa'}(t) u_1(t, 0) \hat{c}^{\dagger}_{\kappa}(0)\rangle$ and $G^{1,h}_{\kappa\kappa'}(\tau, s) = i\Theta(s)\langle \hat{c}_{\kappa}(\tau - s) u_1(\tau - s, \tau) \hat{c}^{\dagger}_{\kappa'}(\tau)\rangle$ corresponding to the transmission amplitude and its complex conjugation, respectively.

By using the Feynman diagram, we obtain

$$G^{1}_{\kappa\kappa'}(\tau, s, t) = \Theta(t)\Theta(s) \sum_{\lambda,q} \int_{-\infty}^{\infty} d\tau' \left[g^{r}_{\kappa'}(\tau - \tau')\right]^{*} \left[M^{\lambda}_{\kappa',\kappa}\right]^{*} e^{-i\omega_{\lambda q}\tau'} \left[g^{r}_{\kappa}(\tau' - \tau + s)\right]^{*} \times \int_{-\infty}^{\infty} dt' g^{r}_{\kappa'}(t - t') M^{\lambda}_{\kappa',\kappa} e^{i\omega_{\lambda q} t'} g^{r}_{\kappa}(t'), \quad (4)$$

where electrons in the conduction band κ emit phonons at t', and holes in the conduction band κ' absorb the emitted phonons at τ' with momentum conservation $\kappa = \kappa' + q$. We define the retarded

bare Green function $g_\kappa^r(t-t') = -i\Theta(t)\langle c_\kappa(t)c_\kappa^\dagger(t')\rangle$, which is evaluated as

$$g_\kappa^r(t-t') = -i\Theta(t)e^{\left(-i\epsilon_\kappa-\frac{\Gamma}{2}\right)(t-t')/\hbar}. \tag{5}$$

The level broadening is defined as $\Gamma = \Gamma_L + \Gamma_R$, where $\Gamma_{L(R)}(\epsilon) = 2\pi\sum_p \left|V_{p\kappa L(p\kappa R)}\right|^2 \delta(\epsilon - \epsilon_{pL(pR)})$, which are energy-independent constants with the wide-band approximation.

The transmission probability $T_\kappa^1(\epsilon_f, \epsilon_i)$, at which incident electrons at κ can tunnel after the interaction with phonon mode λ and momentum exchange q, is expressed in $T_\kappa^1\left(\epsilon_f, \epsilon_i\right) \propto \sum_{\lambda,q} \frac{\Gamma^2\left|M_{\kappa+q,\kappa}^\lambda\right|^2}{\left(\epsilon_f^2+\frac{\Gamma^2}{4}\right)\left(\epsilon_i^2+\frac{\Gamma^2}{4}\right)} \delta\left(\epsilon_i - \omega_{\lambda q} - \epsilon_f\right)$, where the energy of an incident electron is ϵ_i and that of a transmitted electron is ϵ_f after interaction with a phonon. Notice that the energy is conserved by $\epsilon_i - \omega_{\lambda q} = \epsilon_f$ after the incident electron with ϵ_i releases its energy into the phonon with energy $\omega_{\lambda q}$ and tunnels with a reduced energy of $\epsilon_f = \epsilon_i - \omega_{\lambda q}$. We obtain the other terms in the perturbative expansion in a similar manner from the Feynman diagrams in Supplementary Figure 10.

Evaluation of $G_{\kappa\kappa'}^2(\tau, s, t)$ for the second-order peaks in d*G*/d*V*$_b$

When electrons and holes exchange phonons twice, they propagate through intermediate states, e.g., $\kappa \to \kappa_A \to \kappa'$ as in Supplementary Figure 11. Since the transmission amplitude consists of the sum of different intermediate states, quantum interference can appear in the transmission probability. We show that quantum interference appears in $G_{\kappa\kappa'}^2(\tau, s, t)$ between two tunneling processes with a reversed order of phonon interactions. Here, we illustrate the evaluation of $G_{\kappa\kappa'}^2(\tau, s, t) = \Theta(s)\Theta(t)\langle \hat{c}_\kappa(\tau - s)u_2(\tau - s, \tau)\hat{c}_{\kappa'}^\dagger(\tau)\hat{c}_{\kappa'}(t)u_2(t, 0)\hat{c}_\kappa^\dagger(0)\rangle$ with TO(Q) and

LA(M) phonon modes when $\kappa = K$, $\kappa = K'$, $\kappa_A = Q_2$, and $\kappa_B = Q_2'$, as in Figure 3 of the main text:

$$G^2_{KK'}(\tau,s,t) = \Theta(t)\Theta(s)\int_{-\infty}^{\infty} d\tau' \int_{-\infty}^{\infty} d\tau'' \left[g^r_{K'}(\tau-\tau')\right]^* \left(M^{\mathrm{LA}}_{K',Q_2}\right)^* e^{-i\omega_{\mathrm{LA,M}}\tau'} [g^r_{Q_2}(\tau' - \tau'')]^* \left(M^{\mathrm{TO}}_{Q_2,K}\right)^* e^{-i\omega_{\mathrm{TO,Q}}\tau''} [g^r_K(\tau''-\tau+s)]^* \times \int_{-\infty}^{\infty} dt'' \int_{-\infty}^{\infty} dt' g^r_{K'}(t-t'') M^{\mathrm{LA}}_{K',Q_2} e^{i\omega_{\mathrm{LA,M}}t''} g^r_{Q_2}(t'' - t') M^{\mathrm{TO}}_{Q_2,K} e^{i\omega_{\mathrm{TO,Q}}t'} g^r_K(t') + \Theta(t)\Theta(s)\int_{-\infty}^{\infty} d\tau' \int_{-\infty}^{\infty} d\tau'' \left[g^r_{K'}(\tau-\tau')\right]^* \left(M^{\mathrm{TO}}_{K',Q_2'}\right)^* e^{-i\omega_{\mathrm{TO,Q}}\tau'} \left[g^r_{Q_2'}(\tau' - \tau'')\right]^* \left(M^{\mathrm{LA}}_{Q_2',K}\right)^* e^{-i\omega_{\mathrm{LA,M}}\tau''} [g^r_K(\tau''-\tau+s)]^* \times \int_{-\infty}^{\infty} dt'' \int_{-\infty}^{\infty} dt' g^r_{K'}(t-t'') M^{\mathrm{TO}}_{K',Q_2'} e^{i\omega_{\mathrm{TO,Q}}t''} g^r_{Q_2'}(t'' - t') M^{\mathrm{LA}}_{Q_2',K} e^{i\omega_{\mathrm{LA,M}}t'} g^r_K(t') + \Theta(t)\Theta(s)\int_{-\infty}^{\infty} d\tau' \int_{-\infty}^{\infty} d\tau'' \left[g^r_{K'}(\tau-\tau')\right]^* \left(M^{\mathrm{TO}}_{K',Q_2'}\right)^* e^{-i\omega_{\mathrm{TO,Q}}\tau'} \left[g^r_{Q_2'}(\tau' - \tau'')\right]^* \left(M^{\mathrm{LA}}_{Q_2',K}\right)^* e^{-i\omega_{\mathrm{LA,M}}\tau''} [g^r_K(\tau''-\tau+s)]^* \times \int_{-\infty}^{\infty} dt'' \int_{-\infty}^{\infty} dt' g^r_{K'}(t-t'') M^{\mathrm{LA}}_{K',Q_2} e^{i\omega_{\mathrm{LA,M}}t''} g^r_{Q_2}(t'' - t') M^{\mathrm{TO}}_{Q_2,K} e^{i\omega_{\mathrm{TO,Q}}t'} g^r_K(t') + \Theta(t)\Theta(s)\int_{-\infty}^{\infty} d\tau' \int_{-\infty}^{\infty} d\tau'' \left[g^r_{K'}(\tau-\tau')\right]^* \left(M^{\mathrm{LA}}_{K',Q_2}\right)^* e^{-i\omega_{\mathrm{LA,M}}\tau'} [g^r_{Q_2}(\tau' - \tau'')]^* \left(M^{\mathrm{TO}}_{Q_2,K}\right)^* e^{-i\omega_{\mathrm{TO,Q}}\tau''} [g^r_K(\tau''-\tau+s)]^* \times \int_{-\infty}^{\infty} dt'' \int_{-\infty}^{\infty} dt' g^r_{K'}(t-t'') M^{\mathrm{TO}}_{K',Q_2'} e^{i\omega_{\mathrm{TO,Q}}t''} g^r_{Q_2'}(t'' - t') M^{\mathrm{LA}}_{Q_2',K} e^{i\omega_{\mathrm{LA,M}}t'} g^r_K(t') + (\text{the other terms by the rest of the phonon modes}). \quad (6)$$

The transmission probability $T^2_K(\epsilon_f, \epsilon_i)$ from $G^2_{\kappa\kappa'}(\tau,s,t)$ is:

$$T^2_{KK'}(\epsilon_f,\epsilon_i) \propto \Gamma^2 \left|M^{\mathrm{LA}}_{K',Q_2}\right|^2 \left|M^{\mathrm{TO}}_{Q_2,K}\right|^2 \left\{ \frac{1}{\left[(\epsilon_i-\epsilon_K)^2+\frac{\Gamma^2}{4}\right]\left[(\epsilon_i-\omega_{\mathrm{TO,Q}}-\epsilon_Q)^2+\frac{\Gamma^2}{4}\right]\left[(\epsilon_f-\epsilon_K)^2+\frac{\Gamma^2}{4}\right]} + \frac{1}{\left[(\epsilon_i-\epsilon_K)^2+\frac{\Gamma^2}{4}\right]\left[(\epsilon_i-\omega_{\mathrm{LA,M}}-\epsilon_Q)^2+\frac{\Gamma^2}{4}\right]\left[(\epsilon_f-\epsilon_K)^2+\frac{\Gamma^2}{4}\right]} + 2\cos(\gamma) \frac{(\epsilon_i-\omega_{\mathrm{LA,M}}-\epsilon_Q)(\epsilon_i-\omega_{\mathrm{TO,Q}}-\epsilon_Q)+\frac{\Gamma^2}{4}}{\left[(\epsilon_i-\epsilon_K)^2+\frac{\Gamma^2}{4}\right]\left[(\epsilon_i-\omega_{\mathrm{TO,Q}}-\epsilon_Q)^2+\frac{\Gamma^2}{4}\right]\left[(\epsilon_i-\omega_{\mathrm{LA,M}}-\epsilon_Q)^2+\frac{\Gamma^2}{4}\right]\left[(\epsilon_f-\epsilon_K)^2+\frac{\Gamma^2}{4}\right]} \right\} \times \delta\left(\epsilon_i - \omega_{\mathrm{TO,Q}} - \omega_{\mathrm{LA,M}} - \epsilon_f\right) + (\text{the other terms by the rest of the phonon modes}), \quad (7)$$

where $\left|M^{\mathrm{TO}}_{Q_2,K'}\right|^2 = \left|M^{\mathrm{TO}}_{Q_2',K}\right|^2$ and $\left|M^{\mathrm{LA}}_{Q_2',K}\right|^2 = \left|M^{\mathrm{LA}}_{Q_2,K'}\right|^2$ due to time-reversal symmetry. γ is the geometric phase, which equals half of the solid angle Ω covered by geodesics connecting the spin

states of the electrons at K, K', Q_2, and Q_2' valleys. In the presence of time-reversal symmetry, $\gamma = \pi$ (gauge-independently, see Supplementary Figure 11), and $T_K^2(\epsilon_f, \epsilon_i)$ becomes

$$T_{KK'}^2(\epsilon_f, \epsilon_i) \propto \frac{\Gamma^2 \left| M_{K',Q_2}^{\mathrm{LA}} \right|^2 \left| M_{Q_2,K}^{\mathrm{TO}} \right|^2 (\omega_{\mathrm{TO,Q}} - \omega_{\mathrm{LA,M}})^2 \delta(\epsilon_i - \omega_{\mathrm{TO,Q}} - \omega_{\mathrm{LA,M}} - \epsilon_f)}{\left[(\epsilon_i - \epsilon_K)^2 + \frac{\Gamma^2}{4}\right]\left[(\epsilon_i - \omega_{\mathrm{TO,Q}} - \epsilon_Q)^2 + \frac{\Gamma^2}{4}\right]\left[(\epsilon_i - \omega_{\mathrm{LA,M}} - \epsilon_Q)^2 + \frac{\Gamma^2}{4}\right]\left[(\epsilon_f - \epsilon_K)^2 + \frac{\Gamma^2}{4}\right]} +$$

(the other terms by the rest of the phonon modes). (8)

Since the energy scale of phonons, tens of meV, is much smaller than that of the energy gap between band bottoms and the Fermi level, a few eV, the tunneling processes with destructive interference by geometric phase $\gamma = \pi$ hardly contribute to the second-order peaks in $\mathrm{d}G/\mathrm{d}V_{\mathrm{b}}$.

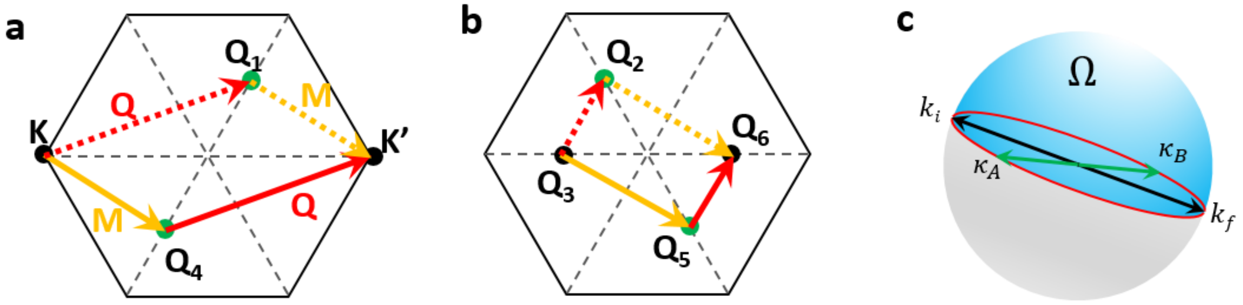

Supplementary Figure 11. Geometric phase and interference trajectory in momentum space. a, b, Scattering routes of tunneling electrons at the K and Q valleys after interacting with M and Q phonons. The two-phonon scattering processes involving M and Q phonons invoke quantum interference effects for all the electrons at the K and Q valleys. **c,** Because of the time-reversal symmetry between k_{i} and k_{f} (κ_{A} and κ_{B}), the solid angle Ω of the geodesic polygon is 2π and the geometric phase becomes π.

Owing to the time-reversal symmetry between K and K' (Q_3 and Q_6) in Supplementary Figure 11a and 11b, the solid angle $\Omega = 2\pi$ (blue shade) and thus the geometric phase is $\gamma = \pi$. Two tunneling processes with a reversed order of phonon interactions (red and blue) exhibit quantum interference.

Discussion on the quantum interference in the second-order inelastic scattering processes

We further elaborate on the non-vanishing quantum interference in the second-order inelastic scattering processes when the spin-momentum locking comes into play, which is indeed the central theme of our data interpretation. According to the common wisdom, virtual processes in quantum mechanical scattering events should cancel their redundant phases through an appropriate gauge transformation. Those virtual processes, evidently distinct from the Aharonov-Bohm effect, should occur in some electron–phonon scattering processes as well. This argument is so powerful and fundamental that there seems no room for any further alternatives. Against all these odds, however, a non-trivial phase can be associated with IETS once the virtual inelastic scattering processes are effectively quenched to a few limited channels and the scattering routes are involved with abrupt turnarounds of spin degrees of freedom, as in the monolayer SC-TMDs. With these restrictions, the non-trivial phase should be emerged because the quenched virtual scattering phase space effectively introduces a specific close path for a certain two-phonon process and the electron spin along the path should rotate following the unique spin-momentum locking in the monolayer SC-TMDs.

We try to elaborate the above statement with the second-order perturbation theory and the Fermi's Golden rule. At first, we should point out that the electron–phonon scatterings and their

strengths are particularly strong at high symmetric points in the SC-TMDs, as discussed in the main text. Accordingly, the electron–phonon scattering phase space should be effectively quenched to the selected scattering processes that involve the phonons at the high symmetric points of Q, M and K. Next, we introduce a simple harmonic perturbation with two distinct phonon modes to capture the essential aspect of the inelastic two-phonon electron scattering processes,

$$V(t) = V_a e^{i\omega_a t} + V_b e^{i\omega_b t} + h.c. \tag{9}$$

Here, $V_{\lambda=a,b}$ denotes the electron–phonon coupling matrix of the phonon modes λ. The harmonic perturbation describes the energy exchanges between the phonons with momenta of q_a and q_b and conducting electrons. We now consider the transition rate $\mathrm{W}_{k_f k_i}$ to describe the electron tunneling with an incident momentum k_i and a transmitted momentum k_f. In particular, we focus on the second-order $\mathrm{W}_{fi}^{(2)}$, which describes two-phonon inelastic electron scattering events with intermediate states *A* and *B*, as illustrated in the figure below (Supplementary Figure 12).

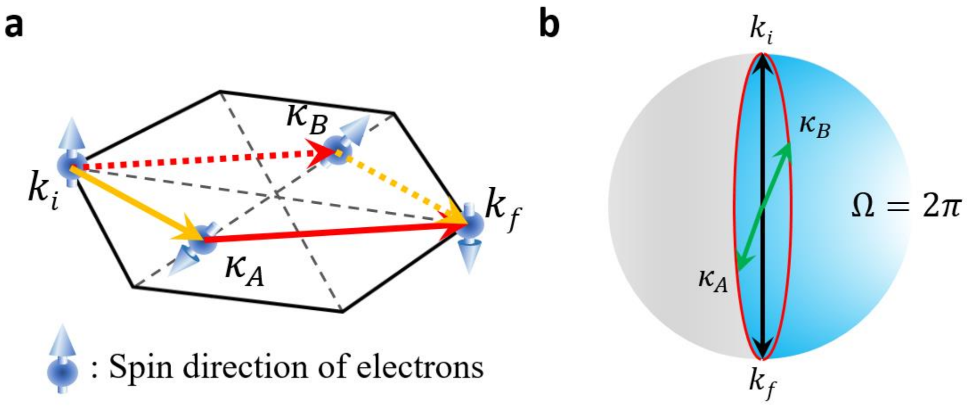

Supplementary Figure 12. a, Inelastic electron–phonon scattering paths of A (solid lines) and B (dotted lines) and the electron spin directions. **b,** The geodesic polygon (red lines) representing electronic spin states of k_i, k_f, κ_A, and κ_B on the Bloch sphere. The solid angle of the

geodesic polygon is $\Omega = 2\pi$ no matter when k_{i} and k_{f} (κ_{A} and κ_{B}) are time-reversal counter partners.

With the Fermi golden rule, the two-phonon IETS processes with the conducting electrons around the conduction-band minimum at Q can be described as

$$W^{(2)}_{k_f k_i} \propto \left| \frac{\langle k_f|V_b|\kappa_A\rangle\langle\kappa_A|V_a|k_i\rangle + \langle k_f|V_a|\kappa_B\rangle\langle\kappa_B|V_b|k_i\rangle}{E_f - E_i - E_A} \right|^2 \delta\big(E_f - E_i - \hbar\omega_a - \hbar\omega_b\big). \quad (10)$$

The momentum conservation law allows the two independent inelastic scattering paths; electrons are scattered along the path-A through a virtual state $|\kappa_{\mathrm{A}}\rangle$, and the path-B through a virtual state $|\kappa_{\mathrm{B}}\rangle$ (Supplementary Figure 12). Here, $|\kappa_{\mathrm{A}}\rangle$ and $|\kappa_{\mathrm{B}}\rangle$ respectively indicate the intermediate states after scatterings with the phonons with q_a and q_b: $\kappa_A = k_i - q_a$ and $\kappa_B = k_i - q_b$. We took into account of the degeneracy of Q-valleys in SC-TMDs by setting $E_A = E_B$, which is one of the important physical traits of the SC-TMDs for the development of quantum interference. The numerator in the equation above could be responsible for plausible quantum interference in IETS spectra, but there seems no rooms for any gauge-invariant phase accumulation in the course of the electrons stopping by the intermediate states $|\kappa_{\mathrm{A,B}}\rangle$ with the interaction $V_{a,b}$. In other words, the intermediate states in the virtual tunneling processes appear as inverted brackets of $|\kappa_{\mathrm{A}}\rangle\langle\kappa_A|$ and $|\kappa_{\mathrm{B}}\rangle\langle\kappa_B|$, and the operators V_a and V_b are shared in the first and the second terms at low temperatures, at which only the phonon emissions of q_a and q_b can be considered. Thus, through an appropriate gauge transformation like

$$\left|\langle k_f|V_b|\kappa_A\rangle\langle\kappa_A|V_a|k_i\rangle + \langle k_f|V_a|\kappa_B\rangle\langle\kappa_B|V_b|k_i\rangle\right|^2$$

$$\rightarrow \left|\langle k_f\big|V_b\big(e^{i\alpha}|\kappa_A\rangle\big)\big(\langle\kappa_A|e^{-i\alpha}\big)V_a\big|k_i\rangle + \langle k_f\big|V_a\big(e^{i\beta}|\kappa_B\rangle\big)\big(\langle\kappa_B|e^{-i\beta}\big)V_b\big|k_i\rangle\right|^2, \quad (11)$$

the different phase factors $e^{i\alpha}$ and $e^{i\beta}$ from the virtual states $|\kappa_A\rangle$ and $|\kappa_B\rangle$ should be vanished and no quantum interference effects are expected in inelastic electron–phonon scattering processes.

However, when the scattering routes involve with the abrupt spin state (η_k) changes introduced by the spin-momentum locking and the inversion symmetry breaking in monolayer SC-TMDs, a non-trivial quantum phase can emerge and consequently influence the two-phonon electron scattering processes, as explicitly observed in our measurements. In monolayer SC-TMDs, the second-order transition rate W_{fi} should then be modified with the spin states η_k:

$$W_{k_f k_i}^{(2)} \propto \left| \frac{\left(\eta_{k_f}^{\dagger}\eta_{\kappa_A}\eta_{\kappa_A}^{\dagger}\eta_{k_i}\right)\langle k_f|V_b|\kappa_A\rangle\langle\kappa_A|V_a|k_i\rangle + \left(\eta_{k_f}^{\dagger}\eta_{\kappa_B}\eta_{\kappa_B}^{\dagger}\eta_{k_i}\right)\langle k_f|V_a|\kappa_B\rangle\langle\kappa_B|V_b|k_i\rangle}{E_f - E_i - E_A} \right|^2 \times \delta\left(E_f - E_i - \hbar\omega_a - \hbar\omega_b\right). \quad (12)$$

The differing spin states at the high symmetry points demand that the inverted brackets should be expressed as $|\kappa_A\rangle\eta_{\kappa_A}\eta_{\kappa_A}^{\dagger}\langle\kappa_A|$, and $\eta\eta^{\dagger}$ is far from the identity matrix for any spinor η. Therefore, the non-trivial phase difference γ is introduced in $W_{k_f k_i}^{(2)}$ as

$$\gamma = \mathrm{Arg}\left[\eta_{k_f}^{\dagger}\eta_{\kappa_A}\eta_{\kappa_A}^{\dagger}\eta_{k_i}\left(\eta_{k_f}^{\dagger}\eta_{\kappa_B}\eta_{\kappa_B}^{\dagger}\eta_{k_i}\right)^{*}\right] = \mathrm{Arg}\left[\eta_{k_f}^{\dagger}\eta_{\kappa_A}\eta_{\kappa_A}^{\dagger}\eta_{k_i}\eta_{k_i}^{\dagger}\eta_{\kappa_B}\eta_{\kappa_B}^{\dagger}\eta_{k_f}\right], \quad (13)$$

where $\mathrm{Arg}[z]$ denotes the phase of a complex number z. Since the spinors appear with their Hermitian conjugates in the closed path, it is straightforward to show that the phase γ is gauge invariant and cannot be eliminated by a gauge transformation. The gauge invariant phase in two-level systems becomes $\gamma = \Omega/2$ and Ω is the solid angle of geodesic polygon enclosed by the spinors on the Bloch sphere (Supplementary Figure 12).

The observable physical content of the solid angle Ω was firstly recognized by Prof. M. Berry in the following paper [Berry, M. V. The adiabatic phase and Pancharatnam's phase for polarized light. *J. Mod. Opt.* **34**, 1401–1407 (1987)]. In particular, when η_{k_i} (η_{κ_A}) is a time-reversal partner of η_{k_f} (η_{κ_B}) , it is straightforward to show $\gamma = \pi$ (equivalently $\Omega = 2\pi$), resulting in destructive quantum interference. Note that some of two-phonon inelastic scattering processes in the SC-TMDs, particularly with the Q and M phonons involve such time-reversal spin-state partners. In comparison, inelastic electron scatterings with K and M phonons do not involve with those spin states. Based on these, IETS spectra relating to the Q and M phonon modes become absent in the monolayer SC-TMDs thanks to the destructive quantum interference, but become prevalent in the bilayer SC-TMDs where the additional quantum phase plays no roles.

Supplementary Table

Two-phonon modes in electron–phonon scattering processes	Energy transferred from electrons to a couple of phonons [meV]	Closed quantum interference loop during electron–phonon scattering processes
TA(Q)+TA(Q)	17.2	Yes
TA(Q)+LA(Q)	22.3	Yes
TA(Q)+LA(M)	24.6	Yes
TA(Q)+LA(K)	24.6	—
LA(Q)+LA(Q)	27.4	Yes
LA(Q)+LA(M)	29.7	Yes
LA(Q)+LA(K)	29.6	—
LA(M)+LA(M)	31.9	Yes
LA(M)+LA(K)	31.9	—
LA(K)+LA(K)	31.9	—
TA(Q)+LO(K)	33.2	—
TA(Q)+LO(Q)	36.1	Yes
TA(Q)+TO(M)	37.1	Yes
LA(Q)+LO(K)	38.3	—
TA(Q)+ZO(Γ)	39.9	—
LA(M)+LO(K)	40.6	—
LA(K)+LO(K)	40.6	—
LA(Q)+LO(Q)	41.2	Yes
TA(Q)+ZO(Q)	42.1	Yes
LA(Q)+TO(M)	42.2	Yes
LA(M)+LO(Q)	43.4	Yes
LA(K)+LO(Q)	43.4	—
LA(M)+TO(M)	44.5	Yes
LA(K)+TO(M)	44.5	—
LA(Q)+ZO(Γ)	45.0	—
LA(Q)+ZO(Γ)	47.2	Yes
LA(M)+ZO(Γ)	47.3	—
LA(K)+ZO(Γ)	47.3	—
LO(K)+LO(K)	49.3	—
LA(M)+ZO(Q)	49.4	Yes
LA(K)+ZO(Q)	49.4	—
LO(K)+LO(Q)	52.1	—
LO(K)+TO(M)	53.1	—
LO(Q)+LO(Q)	55.0	Yes
LO(K)+ZO(Γ)	56.0	—

LO(Q)+TO(M)	56.0	Yes
TO(M)+TO(M)	57.0	Yes
LO(K)+ZO(Q)	58.1	—
LO(Q)+ZO(Γ)	58.8	—
TO(M)+ZO(Γ)	59.8	—
LO(Q)+ZO(Q)	60.9	Yes
TO(M)+ZO(Q)	62.0	Yes
ZO(Γ)+ZO(Γ)	62.7	—
ZO(Γ)+ZO(Q)	64.8	—
ZO(Q)+ZO(Q)	66.9	Yes

Supplementary Table 1. Two-phonon inelastic electron tunneling processes considered in the quantum transport simulations. List of all the two-phonon electron–phonon scattering processes that are considered in the theoretical calculations in Figure 3c and 3d of the main text.
